# Galectin-3 Mediates Tumor Progression in Astrocytoma by Regulating Glycogen Synthase Kinase-3β Activity

**DOI:** 10.3390/cimb45040234

**Published:** 2023-04-19

**Authors:** Hung-Pei Tsai, Chien-Ju Lin, Ann-Shung Lieu, Yi-Ting Chen, Tzu-Ting Tseng, Aij-Lie Kwan, Joon-Khim Loh

**Affiliations:** 1 Division of Neurosurgery, Department of Surgery, Kaohsiung Medical University Hospital, Kaohsiung 807, Taiwan; carbugino@gmail.com (H.-P.T.);; 2 School of Pharmacy, College of Pharmacy, Kaohsiung Medical University, Kaohsiung 807, Taiwan; 3 Department of Pathology, Taichung Tzu Chi Hospital, Buddhist Tzu Chi Medical Foundation, Taichung 427, Taiwan; 4 Department of Surgery, School of Medicine, College of Medicine, Kaohsiung Medical University, Kaohsiung 807, Taiwan; 5 Graduate Institute of Medicine, College of Medicine, Kaohsiung Medical University, Kaohsiung 807, Taiwan; 6 Department of Neurosurgery, University of Virginia, Charlottesville, VA 22903, USA

**Keywords:** astrocytoma, galectin-3, GSK3B

## Abstract

Numerous studies have considered galectin-3 or Glycogen synthase kinase 3 beta (GSK3B) as a potential prognosis marker for various cancers. However, the correlation between the protein expression of galectin-3/GSK3B and the clinical parameters of astrocytoma has not been reported. This study aims to validate the correlation between the clinical outcomes and protein expression of galectin-3/GSK3B in astrocytoma. Immunohistochemistry staining was performed to detect galectin-3/GSK3B protein expression in patients with astrocytoma. The Chi-square test, Kaplan−Meier evaluation, and Cox regression analysis were used to determine the correlation between clinical parameters and galectin-3/GSK3B expression. Cell proliferation, invasion, and migration were compared between a non-siRNA group and a galectin-3/GSK3B siRNA group. Protein expression in galectin-3 or GSK3B siRNA-treated cells was evaluated using western blotting. Galectin-3 and GSK3B protein expression were significantly positively correlated with the World Health Organization (WHO) astrocytoma grade and overall survival time. Multivariate analysis revealed that WHO grade, galectin-3 expression, and GSK3B expression were independent prognostic factors for astrocytoma. Galectin-3 or GSK3B downregulation induced apoptosis and decreased cell numbers, migration, and invasion. siRNA-mediated gene silencing of galectin-3 resulted in the downregulation of Ki-67, cyclin D1, VEGF, GSK3B, p-GSK3B Ser9 (p-GSK3B S9), and β-catenin. In contrast, GSK3B knockdown only decreased Ki-67, VEGF, p-GSK3B S9, and β-catenin protein expression but did not affect cyclin D1 and galectin-3 protein expression. The siRNA results indicated that GSK3B is downstream of the galectin-3 gene. These data support that galectin-3 mediated tumor progression by upregulating GSK3B and β-catenin protein expression in glioblastoma. Therefore, galectin-3 and GSK3B are potential prognostic markers, and their genes may be considered to be anticancer targets for astrocytoma therapy.

## 1. Introduction

Astrocytoma is a common type of brain tumor in humans. Astrocytomas were classified into four grades by the World Health Organization (WHO) [1]. Grade I tumors, such as pilocytic astrocytomas, are benign and slow growing. Grade II tumors comprise relatively slow-growing diffuse astrocytomas. Mitosis has been identified in grade III tumors, and grade III and IV tumors are highly malignant, exemplified by anaplastic astrocytoma and GB. GB is the most common and most aggressive human malignant primary brain tumor. Histopathology demonstrates vascular thrombosis, microvascular proliferation, and necrosis. Despite advancement in surgery, radiotherapy, and chemotherapy of brain tumors, the overall survival of patients with GB remains extremely poor. Moreover, the 5-year survival rate for GB is limited to 5 % [2].

Galectins comprise 16 carbohydrate-binding proteins that bind to β-galactoside moieties [3,4,5,6]. Galectin-3 is one of the Galectin family members and regulates some progression, including cell growth, differentiation, migration, adhesion, angiogenesis, and malignancy transformation, apoptosis, and drug resistance [3,4,5,6,7,8,9,10,11,12]. In breast and prostate cancer, galectin-3 was reported to regulate metastasis via binding to cell adhesion-related molecules and inhibiting cell–cell and cell–ECM interactions [13]. In addition, overexpression of galectin-3 protein was shown to enhance the motility and invasiveness of breast and lung carcinoma cells [14,15]. Khaldoyanidi et al. reported that the breast cancer cells with highly metastatic ability exhibited high levels of galectin-3 expression, which significantly increased adhesion to endothelial cells [16]. In contrast, galectin-3 downregulation decreased the motility of human colon cancer cells and human glioblastomas [17,18]. Although several studies have examined the role of galectin-3 in cancer, there are very few reports on this protein in the context of astrocytoma.

Recently, some reports have identified a correlation between galectin-3 and β-catenin expression in several cancers. Furthermore, galectin-3 mediates the Wnt signaling pathway and nuclear β-catenin accumulation by regulating glycogen synthase kinase-3β (GSK3B) activity in human colon cancer cells [19]. However, the clinicopathological role of GSK3B and galectin-3 in astrocytoma remains unelucidated. Therefore, our study aims to validate not only the relationship between the clinical outcomes but also the protein expression of galectin-3 and GSK3B and the correlation between galectin-3 and GSK3B in astrocytoma. The results of this study may be useful in identifying potential prognostic markers whose genes could be considered as anticancer targets for astrocytoma therapy.

## 2. Materials and Methods

### 2.1. Patients

In total, 113 patients with astrocytoma, from the Neurosurgery Department of Chung-Ho Memorial Hospital, Kaohsiung Medical University, Taiwan, were included in this study. Patients who were diagnosed via biopsies only or had incomplete medical records, no follow-up visits, low-quality pathological results, or poor immunohistochemical staining were excluded.

### 2.2. Immunohistochemistry Staining

For each case, 3-µm sections were cut from tissue blocks of biopsies that were previously fixed with formalin and embedded in paraffin. These sections were deparaffinized, rehydrated, and autoclaved at 121 °C for 10 min in Target Retrieval Solution (pH 6.0; DAKO) to retrieve previously unreactive antigens. Following incubation for at room temperature for 20 min, the endogenous peroxidase in the sections was blocked with 3% hydrogen peroxide at room temperature for 5 min. After washing twice with Tris buffer, the sections were incubated with a 1:200 dilution of the primary antibody (anti-galectin-3 or anti-GSK3B) at room temperature for 1 h. Then, the sections were washed twice with Tris buffer and incubated with a secondary antibody conjugated with horseradish peroxidase for 30 min at room temperature. Finally, the slides were incubated in 3,3’-diaminobenzidine (Dako) for 5 min followed by counterstaining with Mayer’s hematoxylin for 90 sec and mounting with Malinol mounting medium. An immunohistochemical stain scoring system was used to classify the intensity as either low- or high-level expression. Scores representing the proportion of positive-stained tumor cells were graded as 0 (no positive tumor cells), 1 (<10%), 2 (10–50%), and 3 (>50%). The intensity of staining was determined as 0 (no staining), 1 (weak staining), 2 (moderate staining), and 3 (strong staining). Staining index (SI) was calculated as the ratio of intensity: proportion of positive tumor cells, resulting in scores of 0, 1, 2, 3, 4, 6, and 9. We chose 4 as the cutoff value. SI > 4 was indicative of high GSK3B/galectin-3 expression, and <4 was indicative of low GSK3B/galectin-3 expression.

### 2.3. Cell Culture

All the cell lines were incubated at 37 °C with 5% CO_2_. GBM8401 and GBM8901 cells were cultured in Roswell Park Memorial Institute (RPMI) medium including 10% fetal bovine serum (FBS). U87-MG and SVGp12 cell lines were cultured in minimal essential medium including 10% FBS. G5T cells were cultured in Dulbecco’s modified Eagle’s medium including 10% FBS. The GBM8401, GBM8901, U87-MG, and G5T cell lines were originally isolated from patients with GB. SVGp12 was originally isolated from healthy tissue and was used as a control.

### 2.4. Transfection

GSK3B siRNA (si-GSK3B) and galectin-3 siRNA (si-galectin-3) (both 5 µM) were transfected into glioma cells using DharmaFECT transfection reagents (DharmaconTM). Following transfection, the cells were cultured for 3 days before use. Galectin-3 and GSK3B protein expression were detected by western blotting.

### 2.5. Cell Viability

GB cells were reconstituted in the RPMI culture medium containing 10% FBS, and 2 mL (approximately 1 × 10^6^ cells) of the cell suspension was placed in every well of a 6-well plate that was incubated at 37 °C with 5% CO_2_ for 24 h, under saturated humidity. Following co-culture with 5 μM si-GSK3B or si-galectin-3 for 48 h, MTT assay was performed to enumerate viable cells.

### 2.6. Western Blotting

All the 1 × 10^6^ cells were placed in 200 μL lysis buffer. Then, 50 μg protein that was extracted from each sample underwent sodium dodecyl sulfate-polyacrylamide gel electrophoresis at 50 V for 4 h. The protein was transferred from the gel on to a polyvinylidene difluoride membrane. After incubation in blocking buffer for 1 h, the membranes were incubated with the following primary antibodies at room temperature for 2 h: anti-GSK3B (1:500; Abgent; San Diego, CA, USA), anti-galectin-3 (1:500; Abgent), anti-PARP (1:500; Cell Signaling; Danvers, MA, USA), anti-cleaved caspase-3 (1:200; Cell Signaling), anti-Ki-67 (1:1000; Dako, Santa Clara, CA, USA), anti-vascular endothelial growth factor (VEGF) (1:500; Cell Signaling; Danvers, MA, USA), anti-β-actin (1:20,000; Sigma-Aldrich; St. Louis, MO, USA), anti-cyclin D1 (1:500; Thermo Fisher Scientific; Waltham, MA, USA), anti-β-catenin (1:500; Abcam; Cambridge, UK), and anti-p-GSK3B s9 (1:500; Cell Signaling; Danvers, MA, USA). Further, the membranes were incubated with the secondary antibodies, goat anti-Rabbit (1:5000; Millipore; St. Louis, MO, USA) and goat anti-Mouse (1:5000; Millipore; St. Louis, MO, USA), for 90 min. Western Lighting^®^ Plus-ECL solution (Perkin-Elmer; Waltham, MA, USA) was used to detect specific bands utilizing a MiniChemi™ Chemiluminescent Imaging and Analysis System (Sage Creation Science; Beijing, China).

### 2.7. In Vitro Invasion Assay

Cell invasion assays were performed to investigate cell movement in vitro using Corning^®^ Transwell^®^ chambers and Corning^®^ Matrigel^®^ (Sigma-Aldrich). GBM8401 cells were seeded at 5 × 10^5^ per insert. The lower chamber of the Transwell^®^ was filled with 2 mL medium containing nonsense siRNA (non-siRNA), si-GSK3B, or si-galectin-3. Following incubation for 24 h, cells remaining on the upper surface of the Transwell^®^ membrane were removed using a cotton swab. Cells that had invaded the membrane to reach the bottom of the insert were fixed, stained, photographed, and quantified by counting six random high-powered fields.

### 2.8. In Vitro Migration Assay

GBM8401 cells were used to assess cell migration by a wound healing assay culture-insert (ibidi) for six-well plates. The insert was coated with cells seeded at 1 × 10^5^ per insert and cultured at 37 °C for 12 h. si-GSK3B, si-galectin-3, and non-siRNA were added after 24 h. After 1 day, the plates were washed twice with PBS and photographed.

### 2.9. Data Analysis

SPSS 19.0 (IBM Corp.) was used for statistical analysis. The Chi-squared test was performed to determine the correlation between galectin-3/GSK3B protein expression and a specific clinicopathological parameter. Survival rate was analyzed by the Kaplan–Meier method utilizing the log-rank test. Multivariate Cox regression analyses were used to verify the independent effect of each variable; *p* < 0.05 was considered to be statistically significant.

## 3. Results

### 3.1. Correlation between Galectin-3, GSK3B, and Clinical Parameters

**Figure 1:**
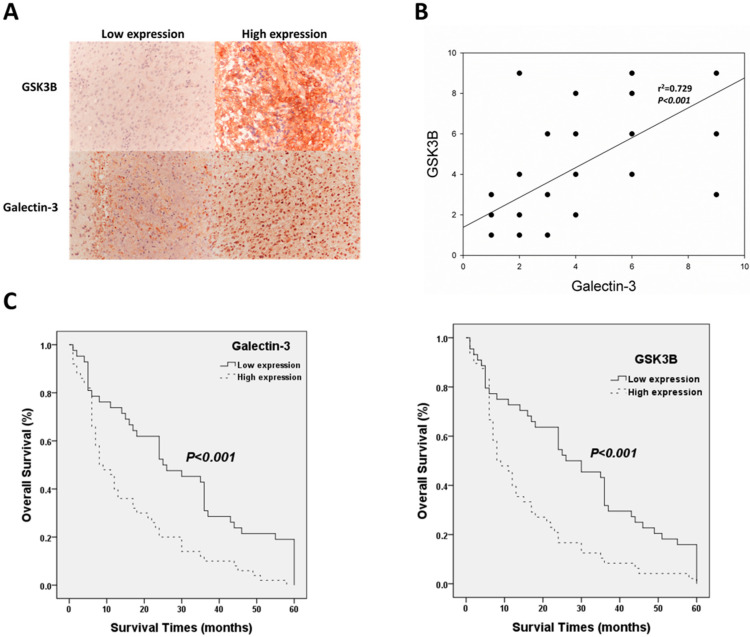
Clinic-pathological role of galectin-3/GSK3B in astrocytoma patients. (**A**) Representative results of immunohistochemical staining for galectin-3/GSK3B using samples obtained from patients with different immunohistochemical staining scores; Magnification is 100×. (**B**) Correlation between galectin-3 and GSK3B expression and survival outcomes. (**C**) Analysis of galectin-3 and GSK3B protein expression using Kaplan−Meier analysis.

Immunohistochemical staining of galectin-3 and GSK3B was analyzed to determine the relationship between protein expression and the clinical parameters of patients with astrocytoma. Figure 1 shows immunohistochemical staining for galectin-3 and GSK3B of sections with low- and high-level expression. GSK3B was expressed in the cytoplasm, and galectin-3 was expressed in the nucleus (Figure 1A). In this study of 113 patients with astrocytoma, 29 were >60 years of age, and 84 were ≤60 years of age. There were 64 males and 49 females. According to the WHO astrocytoma classification, there were 41 and 72 patients with grades II and III/IV astrocytoma, respectively. Of these, 37 cases scored > 70, and 69 scored < 70 according to the Karnofsky performance scale. The Chi-squared test indicated that both galectin-3 (*p* = 0.006) and GSK3B (*p* = 0.007) were significantly associated with the WHO grade (Table 1). Moreover, Spearman’s rank correlation analysis determined that galectin-3 expression was significantly related to GSK3B expression. (*p* < 0.001; Figure 1B). Kaplan−Meier analysis following the log-rank test confirmed the association between galectin-3/GSK3B expression and survival of patients with astrocytoma. The mean survival of patients with high- and low-level galectin-3 expression was 15.68 ± 2.07 and 29.38 ± 3.12 months, respectively. The mean survival of patients with high- and low-level GSK3B expression was 15.21 ± 2.07 and 29.27 ± 3.03 months, respectively. Therefore, high-level galectin-3 and GSK3B expression were significantly associated with poor overall survival (*p* < 0.001 for both) (Figure 1C). Univariate and multivariate analysis revealed that age, WHO astrocytoma grade, galectin-3, and GSK3B were significantly associated with survival time (Table 2). Therefore, galectin-3 and GSK3B were independent prognostic biomarkers in the astrocytoma cases that were studied.

### 3.2. Galectin-3 and GSK3B Protein Expression in Astrocytoma Cells Were Higher Than Those in Normal Cells

**Figure 2:**
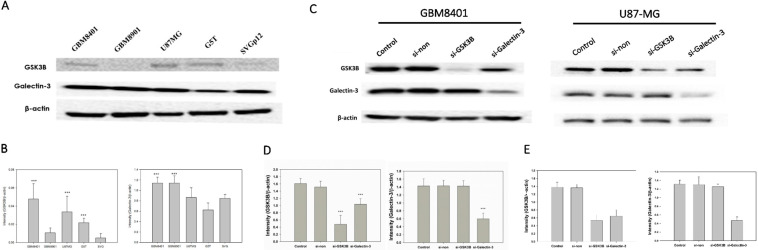
GSK3B and Galectin-3 expression in all GBM cell lines and si-RNA group (*n* = 4). (**A**) Western blotting for GSK3B and galectin-3 expression in all GBM cell lines; (**B**) Relative protein expressions of GSK3B and galectin-3 (*** *p* < 0.001 compared with SVGq12) all GBM cell lines. (**C**) Western blotting for galectin-3 and GSK3B expression between control, non-siRNA, GSK3B siRNA, and galectin-3 siRNA groups in GBM8401 and U87-MG cells. (**D**) Relative protein expression of GSK3B and galectin-3 in GBM8401. *** *p* < 0.001 compared with control group. (**E**) Relative protein expression of GSK3B and galectin-3 in U87-MG. *** *p* < 0.001 compared with control group.

Western blotting was used to investigate the expression levels of galectin-3 and GSK3B proteins in the normal cell line, (SVGp12), and in cancer cell lines (GBM8401, GBM8901, U87-MG, and G5T) (Figure 2A). GBM8401, U87-MG, and G5T cells exhibited a significantly higher expression of GSK3B proteins than that exhibited by SVGq12 (all *p* < 0.001), but no significant difference was observed in expression of GSK3B by GBM8901 cells (Figure 2B). GBM8401 and GBM8901 exhibited significantly higher expression of galectin-3 proteins than did SVGp12 (both *p* < 0.001), but U87MG and G5T did not (Figure 2B).

Of all the cancer cell lines used, GBM8401 and U87MG demonstrated the highest expression level of both galectin-3 and GSK3B. Therefore, we used GBM8401 and U87MG cells in western blotting to analyze galectin-3 and GSK3B protein expression in si-GSK3B and si-galectin-3 groups compared with the control and non-siRNA groups (Figure 2C). GBM8401 and U87MG cells were incubated for 72 h with si-galectin-3, si-GSK3B, or non-siRNA (si-galectin-3 group, si-GSK3B group, and non-siRNA group, respectively). Analysis of western blotting of galectin-3 and GSK3B protein expression involved the comparison of the control and non-siRNA groups with the siRNA groups (Figure 2D,E). The results revealed that knockdown of galectin-3 downregulated the expression of both galectin-3 and GSK3B in GBM8401 and U87MG cells (Figure 2D,E). However, knockdown of GSK3B only downregulated the expression of GSK3B. Furthermore, there was no significant difference observed between the control group and the non-siRNA group (Figure 2D,E).

### 3.3. Silencing GSK3B and Galectin-3 Inhibited GB Cell Proliferation

**Figure 3:**
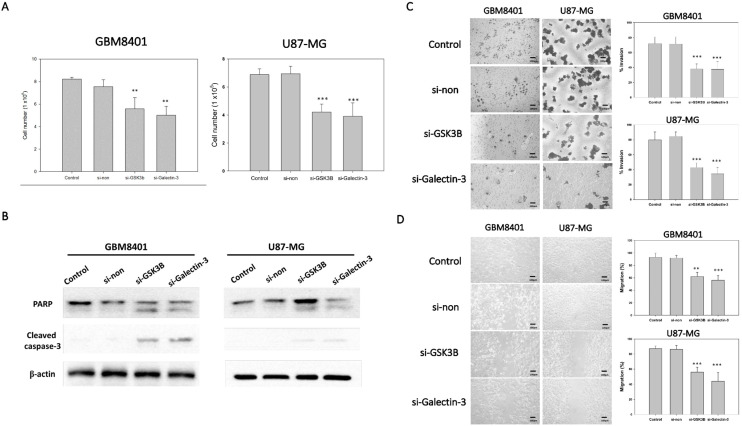
Tumor progression of GBM8401 with galectin-3 or GSK3B siRNA (*n* = 6). (**A**) The cell number of GBM8401 cells cultured for 3 days in 6-well plates following transfection with galectin-3 si-RNA or GSK3B siRNA with MTT assay. (**B**) The protein expressions of PARP and cleaved caspase-3 between control, nonsense siRNA, GSK3B siRNA, and galectin-3 siRNA for 3 days in following transfection with siRNA. (**C**) Transwell invasion assay and relative number of invaded cells in si-GSK3B, si-galectin-3, and non-siRNA groups and control groups following 1 day transfection with siRNA in GBM8401 and U87MG cells. (**D**) Wound healing assay and relative percentage of migration in si-GSK3B, si-galectin-3, non-siRNA, and control groups following 1 day transfection with siRNA in GBM8401 and U87MG cells. ** *p* < 0.01 and *** *p* < 0.001 compared with control group.

To determine the effect of si-galectin-3 or si-GSK3B on cell proliferation, we used an MTT assay to compare the number of viable cells between the siRNA groups with the non-siRNA group. After 72 h incubation with the different siRNAs, cell viability was determined using the MTT assay. The results indicated that the cell viability of the si-galectin-3 group and the si-GSK3B group was significantly lower than that of the control and non-siRNA group in GBM8401 and U87MG cells (Figure 3A). However, no significant differences were observed between the control group and the non-siRNA group (Figure 3A). In addition, we used western blot to detect the PARP and cleaved caspase-3 protein expression. The result showed that both si-galectin-3 and si-GSK3B induced cleaved form in PARP and caspase-3 (Figure 3B). These data implied that the knockdown of galectin-3 and GSK3B correlated with the decreased viability of the astrocytoma cells (Figure 3A,B).

### 3.4. Silencing GSK3B and Galectin-3 Inhibited Invasion and Migration of GB Cells

To demonstrate the ability of invasion and migration, we used Matrigel invasion assay and a wound healing assay. The knockdown of galectin-3 and GSK3B expression with si-galectin-3 and si-GSK3B, respectively, markedly inhibited the invasive (Figure 3C) and migratory (Figure 3D) capabilities of the GB cells. These data suggest that galectin-3 and GSK3B play a role in the invasive and migratory abilities of astrocytoma cells.

### 3.5. Effect of GSK3B and Galectin-3 Silencing on the Protein Expression of β-Catenin, p-GSK3B ser9, VEGF, Ki-67, and Cyclin D1

**Figure 4:**
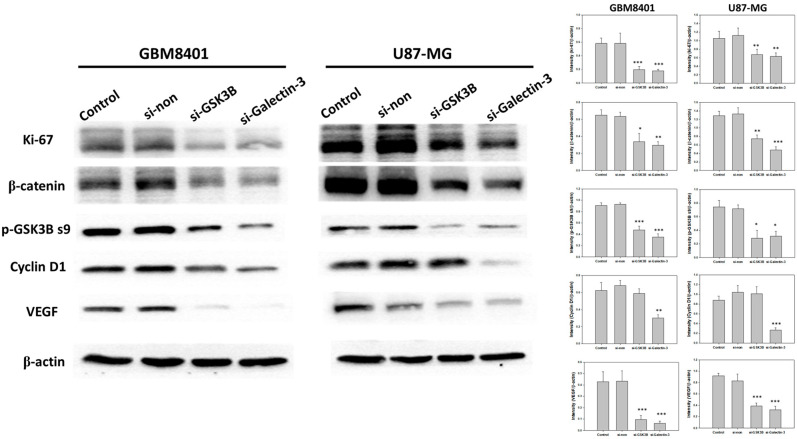
The protein expressions and relative intensity of β-catenin, p-GSK3B ser9, VEGF, Ki-67, and cyclin D1 between control, nonsense siRNA, GSK3B siRNA, and galectin-3 siRNA for 3 days in following transfection with siRNA. (*n* = 6). * *p* < 0.05, ** *p* < 0.01 and *** *p* < 0.001 compared with control group.

To demonstrate the mechanism of the Wnt pathway and tumor progression, a western blot analysis was performed to demonstrate the protein expression of β-catenin, p-GSK3B ser9, VEGF, Ki-67, and cyclin D1 following GSK3B and galectin-3 knockdown in GBM8401 and U87MG cells (Figure 4). The results showed that knockdown of galectin-3 expression significantly downregulated β-catenin, p-GSK3B ser9, VEGF, Ki-67, and cyclin D1 (Figure 4). The knockdown of GSK3B expression downregulated β-catenin, p-GSK3B ser9, VEGF, and Ki-67 but not cyclin D1 (Figure 4). In addition, knockdown of galectin-3 decreased GSK3B protein expression, but knockdown of GSK3B had no effect on galectin-3 protein expression (Figure 2C–E). These data supported that galectin-3 regulated tumor progression by the Wnt pathway.

## 4. Discussion

Galectin-3 binding β-galactoside-specific lectins was found in many species and cell types, and galectin-3 exhibits pleiotropic biologic functions extracellularly, including interacting with the cell surface and extracellular matrix glycoproteins and glycolipids to modulate signaling pathways [20]. Some reports showed that galectin-3 contributes to malignant transformation, tumor cell survival, angiogenesis, and metastasis [21,22,23], and overexpression of galectin-3 protein was closely related to the development of cancers, such as colorectal cancer, breast cancer, melanoma, liver cancer, large-cell lymphoma, brain tumors, and thyroid cancer [24,25]. In our study, galectin-3 protein expression was significantly positively correlated with the World Health Organization (WHO) astrocytoma grade and overall survival time. Multivariate analysis revealed that galectin-3 expression was an independent prognostic factor for astrocytoma. Previous studies have proposed that intranuclear accumulation of galectin-3 regulates the Wnt/β-catenin signaling pathway mainly by transcription activation of cyclin D1, c-myc, and other genes to enhance the expression of its target genes, leading to tumorigenesis and adversely affecting prognosis. In our study, we discovered that knockdown of galectin-3 attenuated cell proliferation, migration, and invasion and inhibited the Ki-67, VEGF, and cyclin D1 protein expression in astrocytoma cells. Ki-67 and cyclin D1 are malignant biomarkers of astrocytoma [26,27].

Previous studies suggested that galectin-3 activates Wnt signaling in human breast cancers [28,29]. Wnt signaling also plays a key role in colon carcinogenesis [30]. The Wnt/β-catenin pathway plays a key role in development, tissue homeostasis, and cancer susceptibility [30,31]. GSK3B is a serine/threonine protein kinase involved in the regulation of protein synthesis, glycogen metabolism, cell proliferation, and survival [32,33]. GSK3B was showed to regulate signaling pathways including Wnt/β-catenin, insulin, Notch, and Hedgehog signaling pathways [34]. GSK3B not only phosphorylates a multitude of metabolic and signaling proteins crucial for cell function, including acetyl-coenzyme- A carboxylase, cyclic adenosine monophosphate-dependent protein kinase, and pyruvate dehydrogenase, but also regulates the intracellular localization and degradation of cyclin D1 [35]. GSK3B is central to a multitude of signaling pathways that regulate a diverse range of cellular functions, from cytoskeletal maintenance [36,37,38] to gene transcription [35,39,40,41,42,43]. When its activation or inhibition is dysregulated, GSK3B has been linked to proliferation, migration, and invasion [44,45,46]. In our study, we found that knockdown of GSK3B attenuated cell proliferation, migration, and invasion and inhibited the β-catenin, p-GSK3B ser9, VEGF, and Ki-67 but not cyclin D1 protein expression in astrocytoma cells. In addition, GSK3B protein expression was significantly positively correlated with the World Health Organization (WHO) astrocytoma grade and overall survival time. Multivariate analysis revealed that GSK3B expression was independent prognostic factors for astrocytoma.

Song et al. showed that galectin-3 modulates β-catenin levels and Wnt signaling by regulating the activity/phosphorylation of GSK3B via PI3K/AKT pathway in colon cancers [19]. However, in our study, Spearman’s rank correlation analysis determined that galectin-3 expression was significantly related to GSK3B expression, and knockdown of galectin-3 decreased β-catenin, p-GSK3B ser9, and GSK3B protein expression, but knockdown of GSK3B had no effect on galectin-3 protein expression. Therefore, GSK3B is upregulated by galectin-3 in astrocytoma.

## 5. Conclusions

In conclusion, our results propose that GSK3B and galectin-3 are involved in important molecular changes that are significantly related to the WHO astrocytoma grade and are independent biomarkers for astrocytoma prognosis. However, knockdown of GSK3B/galectin-3 inhibited cell proliferation, invasion, and migration of astrocytoma cells and galectin-3 mediated tumor progression by upregulating GSK3B protein expression. Both galectin-3 and GSK3B are potential prognostic markers and may be considered as anticancer target genes for astrocytoma therapy.

## Figures and Tables

**Table 1:** Galectin-3/GSK3B expression correlated with clinicopathologic parameters in astrocytomas.

	No.	Galectin-3 Expression		*p*-Value	GSK3B Expression		*p*-Value
		Low	High		Low	High	
Age				0.385			0.517
>60	29	14 (12.4%)	15 (13.3%)		14 (12.4%)	15 (13.3%)	
≤60	84	36 (31.9%)	48 (42.5%)		39 (34.5%)	45 (39.8%)	
Gender				0.202			0.287
Male	64	31 (27.4%)	33 (29.2%)		31 (27.4%)	33 (29.2%)	
Female	49	19 (16.8%)	30 (26.5%)		21 (18.6%)	28 (24.8%)	
WHO Grade				0.006 *			0.007 *
II	41	25 (22.1%)	16 (14.2%)		26 (23%)	15 (13.3%)	
III/IV	72	25 (22.1%)	47 (41.6%)		27 (23.9%)	45 (39.8%)	
Tumor size				0.215			0.521
≤3cm	44	22 (19.5%)	22 (19.5%)		32 (28.3%)	37 (32.7%)	
>3cm	69	28 (24.8%)	41 (36.3%)		21 (18.6%)	23 (20.4%)	
KPS				0.324			0.103
≤70	37	18 (15.9%)	19 (16.8%)		21 (18.6%)	16 (14.2%)	
>70	76	32 (28.3%)	44 (38.9%)		32 (28.3%)	44 (38.9%)	

* Statistically significant (*p* < 0.05).

**Table 2:** Univariate and Multivariate analysis of different prognostic parameters in patients with astrocytoma by Cox-regression analysis.

	Univariate Analysis			Multivariate Analysis		
	Relative Risk	95% CI	*p*	Relative Risk	95% CI	*p*
Age	0.518	0.317–0.847	0.009 *	0.584	0.355–0.959	0.034 *
Gender	1.153	0.762–1.744	0.500			
WHO grade	2.205	1.408–3.454	0.001 *	1.933	1.216–3.071	0.005 *
Tumor size	1.026	0.671–1.567	0.906			
KPS	0.679	0.439–1.051	0.083			
Galectin-3 expression	2.279	1.453–1.051	<0.001 *	2.129	1.348–3.363	0.001 *
GSK3B expression	2.131	1.385–3.279	0.001 *	1.957	1.259–3.042	0.003 *

* Statistically significant (*p* < 0.05).

## Data Availability

Data are contained within the article.
